# Effects of smooth pursuit and second‐order stimuli on visual motion prediction

**DOI:** 10.14814/phy2.14833

**Published:** 2021-05-15

**Authors:** Takeshi Miyamoto, Kosuke Numasawa, Yutaka Hirata, Akira Katoh, Kenichiro Miura, Seiji Ono

**Affiliations:** ^1^ Graduate School of Comprehensive Human Sciences University of Tsukuba Ibaraki Japan; ^2^ Department of Robotic Science and Technology Chubu University College of Engineering Kasugai Japan; ^3^ Department of Physiology Tokai University School of Medicine Kanagawa Japan; ^4^ Department of Pathology of Mental Diseases National Institute of Mental Health National Center of Neurology and Psychiatry Tokyo Japan; ^5^ Graduate School of Medicine Kyoto University Kyoto Japan; ^6^ Faculty of Health and Sport Sciences University of Tsukuba Ibaraki Japan

**Keywords:** eye movement, fixation, time perception, time‐to‐contact task

## Abstract

The purpose of this study was to determine whether smooth pursuit eye movements affect visual motion prediction using a time‐to‐contact task where observers anticipate the exact instant that a partially occluded target would coincide with a stationary object. Moreover, we attempted to clarify the influence of second‐order motion on visual motion prediction during smooth pursuit. One target object moved to another stationary object (6 deg apart) at constant velocity of 3, 4, and 5 deg/s, and then the two objects disappeared 500 ms after the onset of target motion. The observers estimated the moment the moving object would overlap the stationary object and pressed a button. For the pursuit condition, both a Gaussian window and a random dots texture moved in the same direction at the same speed for the first‐order motion, whereas a Gaussian window moved over a static background composed of random dots texture for the second‐order motion. The results showed that the constant error of the time‐to‐contact shifted to a later response for the pursuit condition compared to the fixation condition, regardless of the object velocity. In addition, during smooth pursuit, the constant error for the second‐order motion shifted to an earlier response compared to the first‐order motion when the object velocity was 3 deg/s, whereas no significant difference was found at 4 and 5 deg/s. Therefore, our results suggest that visual motion prediction using a time‐to‐contact task is affected by both eye movements and motion configuration such as second‐order motion.

## Graphical abstract

The purpose of this study was to determine whether smooth pursuit eye movements and motion configuration affect visual motion prediction using a time‐to‐contact task where observers anticipate the exact instant that a partially occluded target would coincide with a stationary object. Our results suggest that visual motion prediction using a time‐to‐contact task is affected by both eye movements and motion configuration such as second‐order motion.

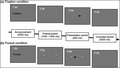

## 1 INTRODUCTION

Smooth pursuit eye movements ensure to hold the image of a moving target on or near the fovea, which allows us to obtain clear vision. In addition, several studies have suggested that an execution of smooth pursuit leads to a change in perception of target motion (Freeman et al., 2010; Spering & Montagnini, 2011). In fact, previous studies have reported some apparent illusions induced by smooth pursuit. As a representative example, the Aubert–Fleischl phenomenon is a well‐known phenomenon where a moving object appears to be moving slower during smooth pursuit compared to fixation (Aubert, 1886; von Fleischl, 1882). Moreover, the Filehne illusion is mentioned as misperception where a stationary background appears to move in the opposite direction to the smooth pursuit direction (Filehne, 1922). Misperception induced by smooth pursuit can be explained by retinal motion images (retinal signal) and efference copies involved in eye movements (extraretinal signal) (Freeman & Banks, 1998; von Holst & Mittelstaedt, 1950; Sperry, 1950). Since smooth pursuit decreases the speed of a pursuit target image on the fovea and increases the speed of a background image in the opposite direction, it is assumed that the visual system cancels the pursuit‐induced retinal image motion in order to maintain perceptual stability. A human study has demonstrated that the visual system compensates the visual perception by integration of retinal and extraretinal signals (Thier et al., 2001). Note that this compensation is usually incomplete due to that both retinal and extraretinal signals incorporate more or less errors (Freeman & Banks, 1998). Therefore, the incomplete compensation during smooth pursuit would lead to different perception of target motion compared to that during fixation.

Those previous studies regarding misperception induced by smooth pursuit raise a question whether smooth pursuit leads to a reduction in the predictive accuracy of visual motion. It is a common situation in daily life and sports activity to predict object motion because the entire trajectory information of the object motion is not always available. Spatial and temporal predictions of visual motion are often estimated by partial information regarding the motion trajectory (Battaglini et al., 2013). Therefore, the accuracy of visual motion prediction could be dependent on how you look at a moving target. In contrast to the misperception induced by smooth pursuit, Spering and colleagues have demonstrated that smooth pursuit provides the better prediction of motion direction than fixation regardless of the retinal motion stimuli. (Spering et al., 2011). For a temporal aspect of visual motion prediction, several studies have used a time‐to‐contact task where observers predict the timing that a partially occluded target would reach an endpoint (Bennett et al., 2010; Yakimoff et al., 1993). Bennett and colleagues have reported that the estimation error is dependent on the occluded duration regardless of the moving target velocity during pursuit, whereas the estimation error is affected by both the occluded duration and moving target velocity during fixation of the arrival position (Bennett et al., 2010). However, since this study has divided the observers into two groups (the pursuit and fixation groups), it is still uncertain whether these eye movements affect visual motion prediction within the individuals. In light of the various factors involved in time perception including both internal and external ones (Matthews & Meck, 2016; Thönes et al., 2018), a within‐subjects design seems to be preferable.

Another question is whether the form of visual motion affects visual motion prediction. It is well documented that smooth pursuit initiation and steady state are altered by motion configuration. A second‐order motion stimulus, which is defined by the combination of a moving Gaussian window in one direction and a stationary background consists of random dots texture, induces poor pursuit responses compared to a first‐order motion stimulus where a Gaussian window and random dots texture move coherently in the same direction (Churan & Ilg, 2001; Hawken & Gegenfurtner, 2001; Ilg & Churan, 2004; Lindner & Ilg, 2000; Miyamoto et al., 2020b). Furthermore, second‐order motion attenuates neuronal activities of the middle temporal (MT) and medial superior temporal (MST) areas (Albright, 1992; Churan & Ilg, 2001; Ilg & Churan, 2004; O’Keefe & Movshon, 1998). In light of the important roles of areas MT and MST in smooth pursuit and motion perception (Newsome et al., 1989; Salzman et al., 1990), the different motion configuration may affect not only the performance of smooth pursuit but also visual motion prediction during smooth pursuit.

Therefore, the purpose of this study was to determine whether smooth pursuit affects visual motion prediction using a time‐to‐contact task where observers anticipate the exact instant that a moving object coincides with a stationary object. In addition, we attempted to apply the second‐order motion stimulus to clarify the influence of motion configuration on visual motion prediction during smooth pursuit.

## 2 MATERIALS AND METHODS

To evaluate a predictive performance, we used a time‐to‐contact task where either of two objects moved toward the other during the presentation period and then both disappeared simultaneously 500 ms after the onset of target motion. The observers estimated the moment the moving object would coincide and overlap the stationary object (both occluded) and pressed a button held in their hands.

### 2.1 Observers

The observers were 12 adults (3 women and 9 men; mean age: 23.5 ± 1.2 years old) and they reported having normal or corrected to normal vision and no known motor deficits. The observers were neither diagnosed with stereoscopic problem nor strabismus. All the observers gave written informed consent in accordance with the Declaration of Helsinki. All the protocols were approved by the Research Ethics Committee at the Faculty of Health and Sport Sciences, University of Tsukuba.

### 2.2 Apparatus and visual stimuli

The observers were seated 57 cm in front of a CRT monitor (22‐inch, RDF223G, Mitsubishi, refresh rate of 60 Hz, spatial resolution of 800 × 600 pixels, background mean luminance 60 cd/m^2^) with head stabilized by a chin rest and a forehead restraint. The observers grasped the button in their dominant hands, which detected the moment of the observers’ manual response. Eye movements from the right eye were detected using a video‐based eye tracking system in which eye position signals are detected from reflected images of the infrared light on the cornea and a black image of the pupil captured by an infrared camera (GS3‐U3‐41C6NIR, FLIR systems Inc.) (Matsuda et al., 2017; Miyamoto et al., 2020b; Ono et al., 2019). The eye position signals were digitized at 1 kHz with 16‐bit precision using CED‐Micro 1401 hardware (Cambridge Electronic Designs, Cambridge, England). Prior to the task, eye position signals from the right eye were calibrated by requiring the observers to fixate a target spot (diameter of 0.3 deg) at known horizontal and vertical eccentricities in binocular viewing condition.

The objects were random dots texture (each dot, 3 × 3 pixels) whose contrast was modulated by a Gaussian window (SD: 0.4 deg) on uniform gray background. Dots had a density of 50% and dot lifetime was equal to presentation duration (500 ms). Although the general time‐to‐contact task uses a small spherical target and a thin striated endpoint, we applied the same form objects (i.e., the combination of Gaussian window and random dot texture) to both the moving and stationary objects in order to be a consistent retinal stimulation for all the conditions.

**FIGURE 1:**
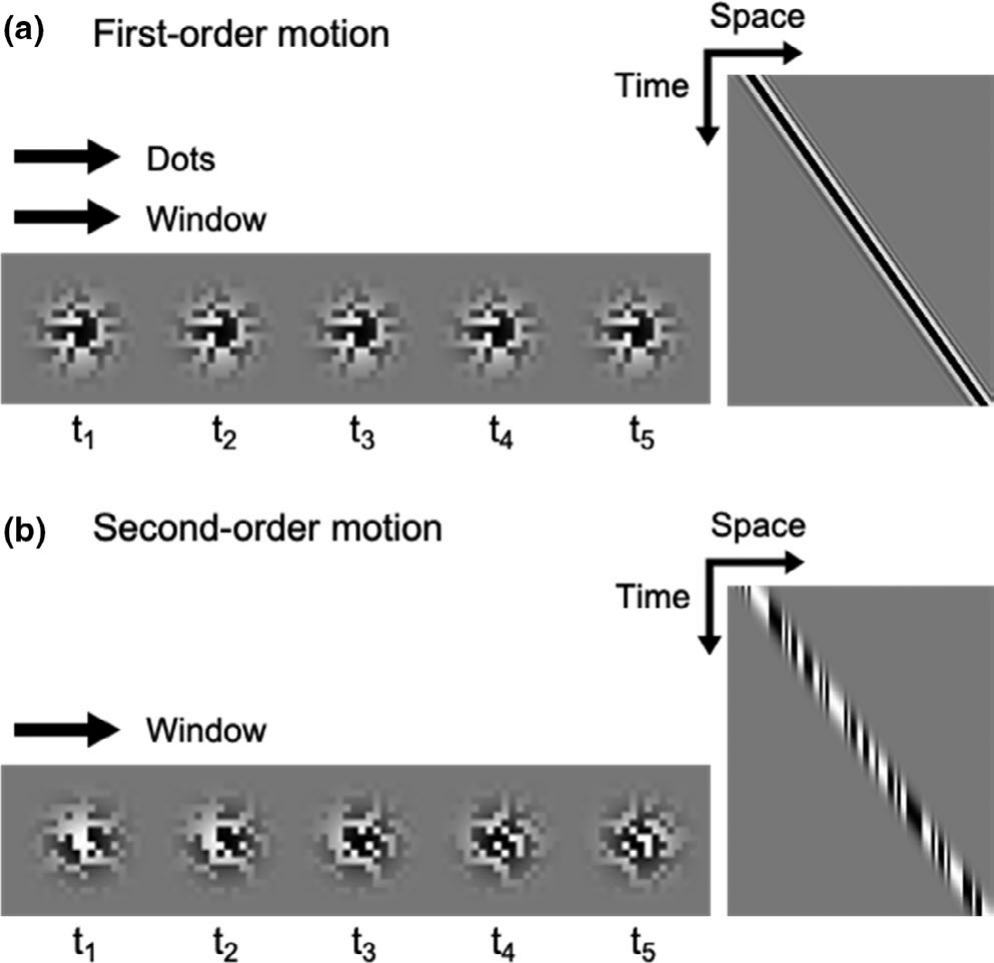
Space—time diagrams of motion stimuli. (a) For the first‐order motion, both the Gaussian window and random dots texture moved in the same direction at the same speed. (b) For the second‐order motion, the Gaussian window moved over a static‐background that consists of random dots texture. Gaussian windows from t_1_ to t_5_ in each visual motion indicate the same target at different points in time within a trial

To test the influence of motion configuration on visual motion prediction during smooth pursuit, we applied the first‐ and second‐order motion stimuli to the moving object. For the first‐order motion, both the Gaussian window and random dots texture moved in the same direction at the same speed (Figure 1a) (Miyamoto et al., 2020a). For the second‐order motion, the Gaussian window moved over a static‐background that consists of random dots texture (Figure 1b) (Chubb & Sperling, 1988; Miyamoto et al., 2020b). All the visual stimuli were generated by Psychophysics Toolbox extensions on MATLAB (Mathworks).

### 2.3 Experimental procedure

The distance between the moving and stationary objects was 6 deg in all the trials, and the moving target velocity was set at 3, 4, and 5 deg/s. The presentation period was 500 ms. The trials consisted of three conditions (fixation, pursuit first‐order, and pursuit second‐order conditions). The observers either tracked or fixated on the left object with their eyes in all the trials regardless of the conditions to be a consistent retinal stimulation (Spering, Pomplun, et al., 2011). For example, the left object was stationary while the right object moved leftward for the fixation condition, whereas only the left object moved rightward for the pursuit first‐ and second‐order conditions. Furthermore, a step‐ramp paradigm (Rashbass, 1961) was applied to both the pursuit and fixation conditions for the moving target. The size of the step was adjusted according to the object velocity so that the moving object would reach the initial presented position in 100 ms.

**FIGURE 2:**
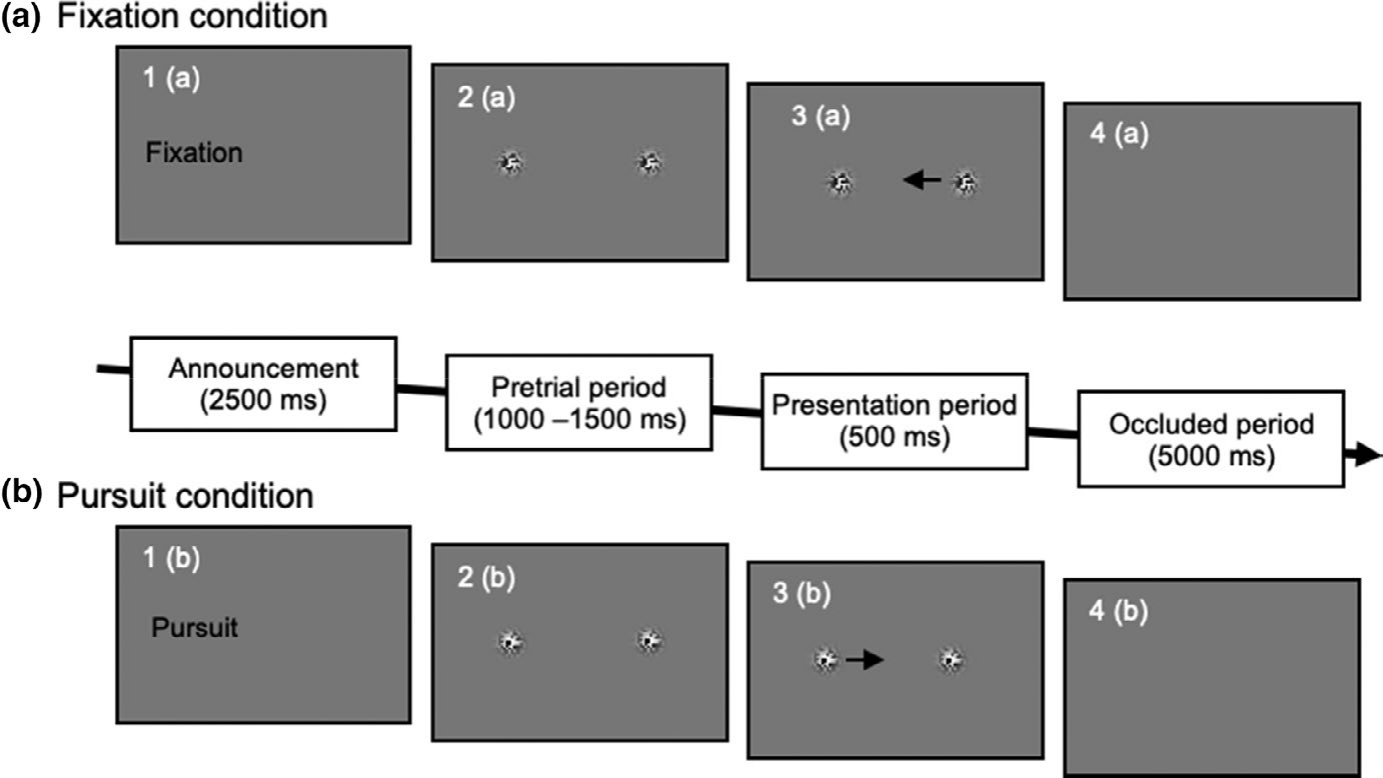
Sequence of the events in each trial. (a) Upper and (b) lower sequences indicate the fixation and pursuit conditions, respectively. The observers gazed the left object in all the trials regardless the conditions; they tracked the left moving object for the pursuit condition, whereas fixated on the left stationary object for the fixation condition. Before the two objects coincided, the two objects disappeared 500 ms after the onset of visual motion. The observers estimated the moment the moving object would coincide and overlap the stationary object (both occluded) and pressed the button held on their hand

Figure 2 represents the full screen configuration and the sequence of the events in each trial. At the beginning of each trial, the word of “fixation” or “pursuit” was presented at the position of 3 deg left from the center for 2500 ms, which informed the eye movement condition (fixation or pursuit) of the trial. Then, two objects appeared at the position of 3 deg left and right from the center. The observers were instructed to fixate on the left object regardless of the conditions. After the fixation of 1000–1500 ms (pretrial period), either of the two objects started to move according to the conditions. The observers must gaze at the left object, and they fixated on the stationary object for the fixation condition or tracked the moving object with their eyes for the pursuit conditions. The two objects were presented for 500 ms after the onset of moving, then made to disappear simultaneously. The observers estimated the moment the moving object would coincide and overlap the stationary object and pressed the button held on their hand. Each trial was initiated at 5000 ms intervals after the two objects disappeared in the last trial. The experimental session consisted of 9 possible combinations: three visual conditions (fixation, pursuit first‐order, and pursuit second‐order conditions) and three types of the object velocity (3, 4 and 5 deg/s). Twenty trials were performed for each combination (total 180 trials) and all the combinations were randomly interleaved. The experimental session was divided into five blocks and 5 min intervals were set between the blocks. The observers were not provided any performance feedback throughout the experiment.

The constant error and absolute error between the correct timing and the observers’ response was calculated for each trial and averaged for each condition. A negative constant error indicated that the observers’ response was earlier compared to the correct timing, and vice versa for a positive constant error. Moreover, the variable error was defined as the standard deviations of the constant error for each visual condition. The variable error indicated the variability of the observers’ response regardless of the accuracy of visual motion prediction. Next, we considered the effect of the previous trial, since several studies have suggested that velocity perception in a current trial (n) is biased by the last trial (n–1) (De Lussanet et al., 2001; Makin et al., 2008). To address this point, we analyzed the three types of data set (3 velocities in the last trial) separately and compared them. As the result of comparison of the constant error among nine possible subdivisions (3 object velocities in the current trial × 3 object velocities in the last trial) for each visual condition, neither significant interaction nor main effect of the last trial was found (summarized in Table 1). Therefore, we determined that the previous trials did not affect the current trial in this study.

**TABLE 1:** Statistical results regarding an effect of object velocity in the last trial (*n*–1) on the constant error in the current trial (*n*)

Condition	Interaction (current trial × last trial)			Main effect of last trial		
	*F*	*p*	*η*_p_^2^	*F*	*p*	*η*_p_^2^
Fixation	1.41	0.25	0.11	< 0.01	0.99	< 0.01
Pursuit first‐order	0.30	0.88	0.03	0.04	0.96	< 0.01
Pursuit second‐order	0.11	0.98	0.01	0.35	0.71	0.03

### 2.4 Data analysis

In the fixation condition, the trials including more than ±2 degrees of eye movement during the presentation period were excluded. In the pursuit conditions, the trials including saccades to the stationary object during the presentation period were excluded. In addition to these criteria, the trials including blinks during the presentation period were also excluded for both conditions. Consequently, 2019 (93.5%) trials out of a total of 2160 trials were used for the analysis. Eye velocity and acceleration were generated by digital differentiation of the position arrays using a central difference algorithm in MATLAB (Mathworks). Velocity and acceleration data were filtered using an 80‐point finite impulse response (FIR) digital filter with a passband of 30 Hz. Saccades were identified and then replaced to NaN according to the criteria of velocity of 30 deg/s or acceleration of 1000 deg/s^2^ before averaging data. Eye velocity traces were aligned on the onset of object motion and averaged with each condition. Pursuit initiation during step‐ramp tracking was taken as the time that average eye velocity reached >3 SD above the pretrial values during fixation. Initial acceleration on smooth pursuit was determined as a mean value in the first 100 ms period of smooth pursuit (Ono et al., 2019; Ono & Mustari, 2007, 2012). Latency on pursuit initiation was defined by the time lapse between the onset of object motion and onset of smooth pursuit. Moreover, we evaluated the saccade rate (frequency) to the total duration of all the trials for the pursuit first‐ and second‐order conditions.

### 2.5 Statistical analysis

We set two comparisons to examine the effects of visual strategies and motion configuration on visual motion prediction. First, we compared the constant error, absolute error, and variable error using a two‐way repeated‐measures analysis of variance (ANOVA) with factors of the visual strategy (2 levels: fixation and pursuit first‐order conditions) and object velocity (3 levels: 3, 4, and 5 deg/s) to clarify whether smooth pursuit affects visual motion prediction. Second, we compared the constant error, absolute error, variable error, pursuit latency, initial eye acceleration, and steady‐state eye velocity using a two‐way repeated‐measures ANOVA with factors of motion configuration (2 levels: pursuit first‐ and second‐order conditions) and object velocity (3 levels) to clarify whether the motion configuration affects visual motion prediction during smooth pursuit. Significant results on the ANOVA were followed up with post‐hoc multiple comparison with Bonferroni correction. Effect sizes of ANOVA were reported as partial *η* ^2^. Partial *η* ^2^ were defined as for small when partial *η* ^2^ < 0.1, moderate when 0.1≤ partial *η* ^2^ < 0.08, large when 0.08≤ partial *η* ^2^ < 0.2, and very large when 0.5≤ partial *η* ^2^, respectively.

Then, we used within‐subjects correlation coefficient, which is a method focusing on the changes of variable within each observer (Bland & Altman, 1995; Miyamoto et al., 2020b), to clarify the relationship between the pursuit responses (i.e., pursuit latency, initial eye acceleration and steady‐state eye velocity) and the constant error, absolute error, and variable error of visual motion prediction. This method treats individual observer as a categorical factor and applies it to multiple regression. All statistical tests were executed with a significance level of 0.05 and conducted by IBM SPSS software version 26 (SPSS Inc.).

## 3 RESULTS

### 3.1 Fixation versus pursuit

**FIGURE 3:**
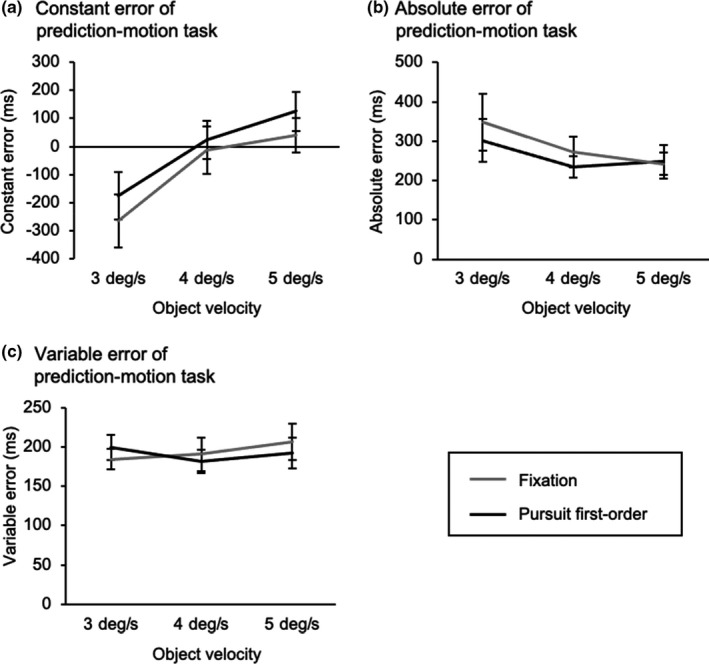
Comparisons of visual motion prediction between the fixation condition (gray lines) and pursuit first‐order condition (black lines). (a) Constant error, (b) absolute error, and (c) variable error are shown as the mean values of individuals. A negative constant error indicates that the observers’ response was early compared to the correct timing, and vice versa for a positive constant error. Error bars indicate standard error of the mean (SEM)

For the constant error (Figure 3a), a significant interaction (visual strategy × object velocity) was not observed (*F*_2,22_ = 2.02, *p* = 0.16, partial *η* ^2^ = 0.16). However, a significant main effect of the visual strategy (*F*_1,11_ = 9.48, *p* = 0.01, partial *η* ^2^ = 0.46) revealed that the constant error for the pursuit first‐order condition was larger than the fixation condition, indicating that the observers showed a delayed response for the pursuit first‐order condition compared to the fixation condition. Moreover, a significant main effect of object velocity (*F*_2,22_ = 28.93, *p* < 0.01, partial *η* ^2^ = 0.72) was found, and post‐hoc test showed that the significant differences of the constant error were observed between all the object velocity.

For the absolute error (Figure 3b), a significant interaction (visual strategy × object velocity) was observed (*F*_2,22_ = 4.68, *p* = 0.02, partial *η* ^2^ = 0.30). Post hoc analysis showed that the absolute error for the pursuit first‐order condition was smaller than the fixation condition at an object velocity of 3 deg/s. At object velocities of 4 and 5 deg/s, no differences were found between the conditions.

For the variable error (Figure 3c), there were neither interaction (*F*_2,22_ = 0.83, *p* = 0.45, partial *η* ^2^ = 0.07) nor main effect (visual strategy: *F*_1,11_ = 0.25, *p* = 0.63, partial *η* ^2^ = 0.02; object velocity: *F*_2,22_ = 0.61, *p* = 0.55, partial *η* ^2^ = 0.05).

### 3.2 First‐order motion versus second‐order motion

**FIGURE 4:**
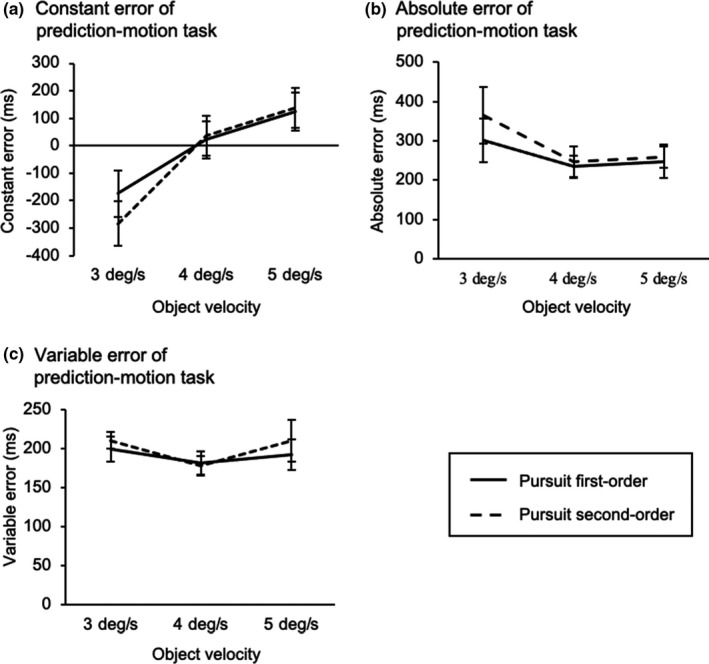
Comparisons of visual motion prediction between the pursuit first‐order condition (solid line) and pursuit second‐order condition (dashed line). (a) Constant error, (b) absolute error, and (b) variable error are shown as the mean values of individuals. A negative constant error indicates that the observers’ response was early compared to the correct timing, and vice versa for a positive constant error. Error bars indicate standard error of mean (SEM)

For the constant error (Figure 4a), a significant interaction (motion configuration × object velocity) was observed (*F*_2,22_ = 11.40, *p* < 0.01, partial *η* ^2^ = 0.51). Post hoc analysis showed that the constant error for the pursuit first‐order condition was smaller (closer to zero) than the pursuit second‐order condition at an object velocity of 3 deg/s. Such a difference was not found for object velocities of 4 and 5 deg/s. Moreover, the significant differences of the constant error were observed between all the object velocity regardless of motion configuration. However, the absolute error (Figure 4b) showed neither interaction (*F*_2,22_ = 1.26, *p* = 0.30, partial *η* ^2^ = 0.10) nor main effect (motion configuration: *F*_1,11_ = 0.46, *p* = 0.06, partial *η* ^2^ = 0.29; object velocity: *F*_2,22_ = 1.02, *p* = 0.38, partial *η* ^2^ = 0.09). Similarly, for the variable error (Figure 4c), there were neither interaction (*F*_2,22_ = 0.69, *p* = 0.52, partial *η* ^2^ = 0.06) nor main effect (motion configuration: *F*_1,11_ = 0.74, *p* = 0.41, partial *η* ^2^ = 0.06; object velocity: *F*_2,22_ = 2.14, *p* = 0.13, partial *η* ^2^ = 0.17).

**FIGURE 5:**
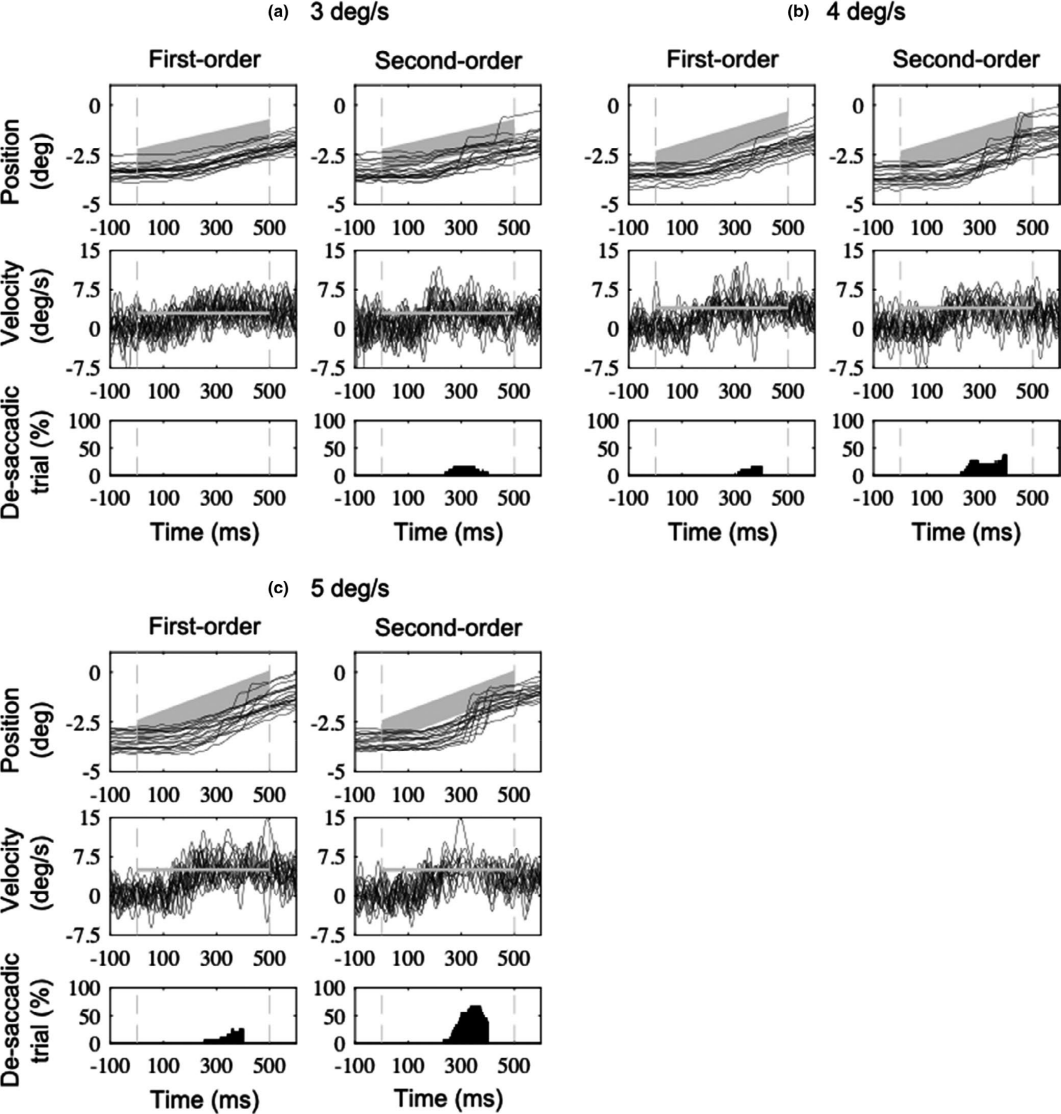
Individual traces of pursuit eye position (top panels) and de‐saccadic eye velocity (middle panels) as a function of time performed by a representative observer for each condition. The bottom panels indicate histograms of detected saccades. Vertical gray dashed lines in all the panels indicate the onset (0 ms) and offset (500 ms) of the visual target. Shaded areas in the top and middle panels indicate the target position (target size: 2 deg), and horizontal shaded lines in the middle panels indicate the target velocity. Upward deflections show rightward eye motion

**FIGURE 6:**
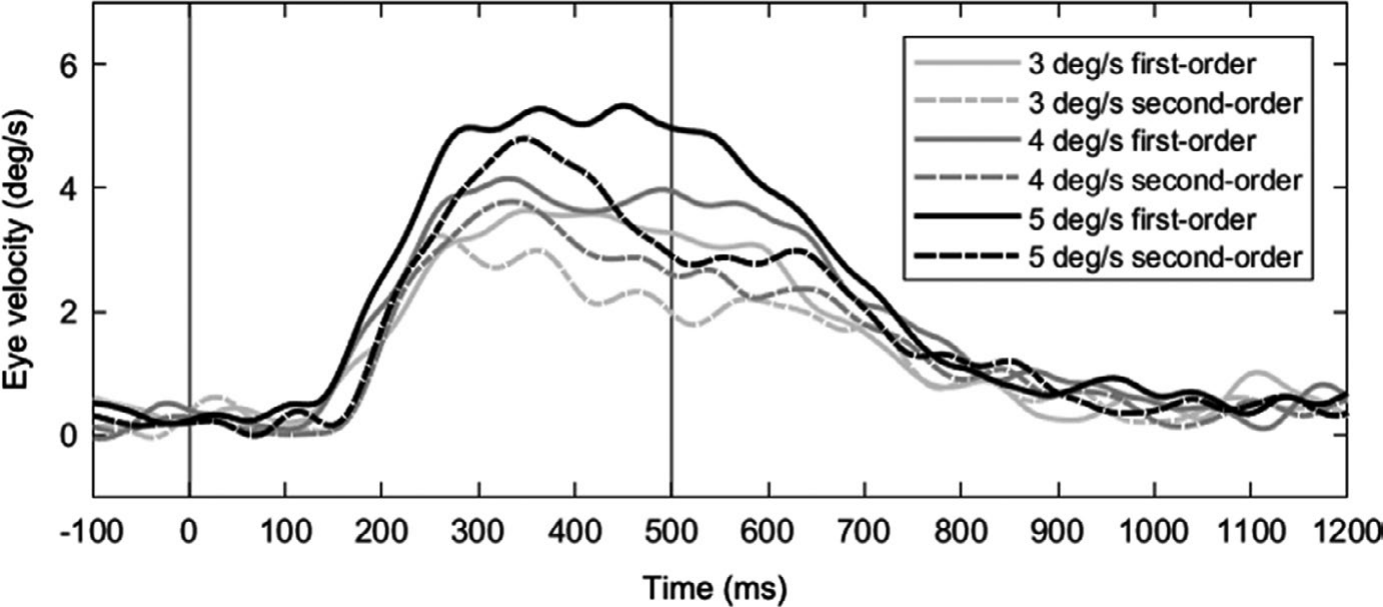
Typical averaged traces of de‐saccadic eye velocity as a function of time performed by a representative observer for each condition. Vertical gray lines indicate the onset (0 ms) and offset (500 ms) of the presentation period. Upward deflections show rightward eye motion

**FIGURE 7:**
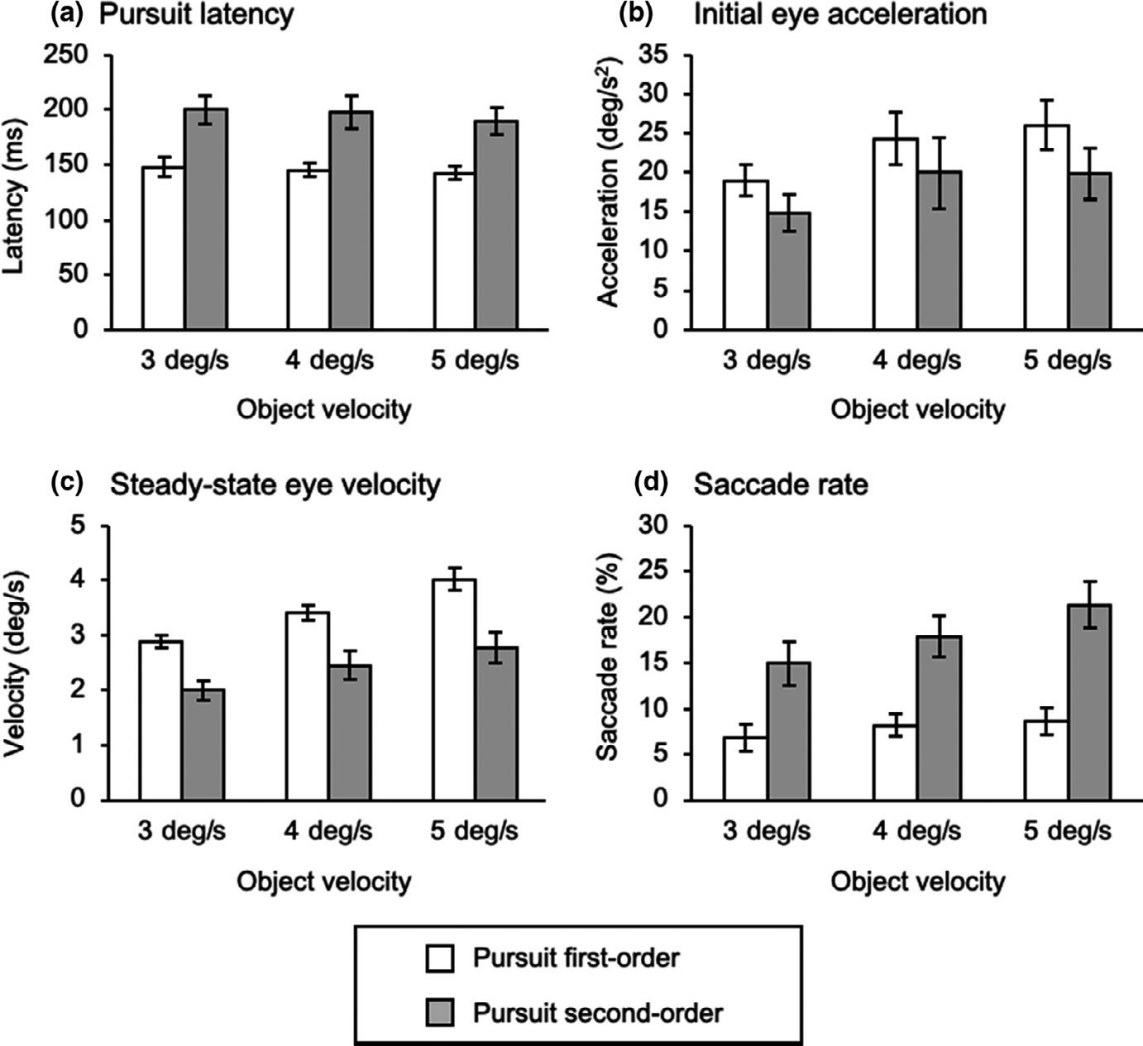
Comparisons of smooth pursuit behavior between the first‐ and second‐order conditions. (a) Pursuit latency, (b) initial eye acceleration, (c) steady‐state eye velocity, and (d) saccade rate are shown as the mean values. Error bars indicate standard error of mean (SEM)

Figure 5 shows typical traces of pursuit eye position, velocity, and the saccade rate as a function of time performed by a representative. Figure 6 shows averaged traces of de‐saccadic eye velocity as a function of time from a representative observer. The properties of smooth pursuit are summarized in Figure 7. There were no interactions in the pursuit latency (*F*_2,22_ = 0.13, *p* = 0.88, partial *η* ^2^ = 0.01), initial eye acceleration (*F*_2,22_ = 0.17, *p* = 0.84, partial *η* ^2^ = 0.02), and steady‐state eye velocity (*F*_2,22_ = 2.93, *p* = 0.07, partial *η* ^2^ = 0.21), and saccade rate (*F*_2,22_ = 3.27, *p* = 0.06, partial *η* ^2^ = 0.23). However, significant main effects of motion configuration were observed in pursuit latency (*F*_1,11_ = 25.52, *p* < 0.01, partial *η* ^2^ = 0.70), initial eye acceleration (*F*_1,11_ = 5.76, *p* = 0.04, partial *η* ^2^ = 0.34), and steady‐state eye velocity (*F*_1,11_ = 43.60, *p* < 0.01, partial *η* ^2^ = 0.80), and saccade rate (*F*_1,11_ = 51.36, *p* < 0.01, partial *η* ^2^ = 0.82). The second‐order motion stimulus led to the longer pursuit latency, smaller eye acceleration/velocity, and the increase in saccade rate than the first‐order motion, indicating second‐order motion stimulus elicited the poor pursuit responses. For the steady‐state eye velocity, a main effect of object velocity was found (*F*_2,22_ = 39.42, *p* < 0.01, partial *η* ^2^ = 0.78), and post hoc test showed the differences in eye velocity between all the object velocity. Although the initial eye acceleration was seen to increase in accordance with the object velocity as well, a statistical main effect was not shown (*F*_2,22_ = 2.75, *p* = 0.08, partial *η* ^2^ = 0.20). The pursuit latency was consistent regardless of the object velocity (*F*_2,22_ = 0.99, *p* = 0.39, partial *η* ^2^ = 0.08). The saccade rate showed a main effect of object velocity (*F*_2,22_ = 39.42, *p* < 0.01, partial *η* ^2^ = 0.78), indicating that the saccade rate at an object velocity of 3 deg/s was smaller than that of 4, 5 deg/s.

For the relationship between the changes in pursuit responses and the three types of error (constant error, absolute error, and variable error) in the time‐to‐contact task, a significant within‐subjects correlation coefficient was obtained between the steady‐state eye velocity and the constant error at an object velocity of 3 deg/s (*r* = 0.69, *p* = 0.01). However, this relationship was not found at object velocities of 4 and 5 deg/s. Furthermore, the absolute error and variable error did not show any significant relationships to the pursuit responses.

## 4 DISCUSSION

In this study, we attempted to determine whether smooth pursuit affects visual motion prediction using a time‐to‐contact task. The results showed that the constant error for the pursuit condition shifted to a later response compared to the fixation condition. Furthermore, we applied second‐order motion to the moving object during smooth pursuit to clarify whether motion configuration affects visual motion prediction. The comparison between the first‐ and second‐order conditions showed that motion configuration affects the constant error when the object was moving at 3 deg/s but not at 4 and 5 deg/s. The constant error for the second‐order motion shifted to an earlier response compared to the first‐order motion. Moreover, this shift to the earlier prediction time induced by the second‐order motion was correlated with the degree of decline in the pursuit velocity for an object velocity of 3 deg/s.

The change in the constant error with object velocity indicates that the lower object velocity leads to an earlier response compared to the higher object velocity, which is comparable with previous studies (Bennett et al., 2010; Yakimoff et al., 1993). It is possible that this change in the constant error could be due to the fact that the observers simply use a constant reaction time strategy. Therefore, we calculated the reaction time between the occlusion onset and the observers’ response to confirm whether the reaction time is constant regardless of the object velocity. As a result, a two‐way ANOVA (3 conditions × 3 object velocities) showed a significant interaction (*F*_4,44_ = 5.89, *p* < 0.01, partial *η* ^2^ = 0.35) and post hoc tests showed that the reaction time became shorter as the object velocity increased for each condition (Table 2). This result confirms that the observers predict the contact time based on the visual motion velocity rather than using a constant reaction time strategy.

**TABLE 2:** The reaction time between the occlusion onset and the observers’ response

	3 deg/s	4 deg/s	5 deg/s
Fixation	1335.9 ms [94.8]	1086.4 ms [83.8]	839.3 ms [60.9]
Pursuit first‐order	1425.1 ms [84.8]	1122.2 ms [68.1]	924.7 ms [68.8]
Pursuit second‐order	1316.7 ms [81.7]	1136.3 ms [73.9]	937.9 ms [72.7]

Values in brackets indicate standard error of mean (SEM).

### 4.1 Influence of eye movements on visual motion prediction

A difference of the constant error between smooth pursuit and fixation has been suggested by previous studies (Bennett et al., 2010; Peterken et al., 1991), and our results also supported this hypothesis by direct comparison within the same observers. For example, the smooth pursuit condition led to a shift of the constant error to a later response compared to the fixation condition. Although the continuous smooth tracking of an occluded moving object has been argued as one of the mechanisms that induces a difference between pursuit and fixation (Lyon & Waag, 1995; Rosenbaum, 1975), subsequent studies have demonstrated that the ocular response after the disappearance of an object does not reflect the continuous tracking (Benguigui & Bennett, 2010; Bennett et al., 2010). In fact, such a residual eye velocity after an object disappeared does not occur without an expectation of object reappearance (Missal & Heinen, 2017). In this study, the results showed a decline in eye velocity after the disappearance of the objects (Figure 6). In addition, since we set the visual stimuli to be a consistent retinal stimulation for the fixation and pursuit first‐order conditions, there is little difference in physical properties between the conditions. Considering that visual motion prediction is estimated by visual information before the disappearance of the moving object (Battaglini et al., 2013), the perceptual aspect such as velocity perception of visual motion could be associated with difference in eye movements.

Signal resources involved in motion perception are different between pursuit and fixation. During fixation, velocity perception is estimated by only the retinal motion signal. In contrast, during smooth pursuit, extraretinal signals, which include the efference copy (von Holst & Mittelstaedt, 1950; Sperry, 1950) and proprioception of the ocular muscle (Velay et al.,^1995^, ^1997^), were important to perceive the object velocity due to a decrease in retinal motion signals. It is well known that this difference leads to a change in motion perception (Thier et al., 2001). Indeed, the shift toward the late prediction time could be due to the change in motion perception induced by smooth pursuit, in accordance with the Aubert–Fleischl phenomenon where a moving object appears to be moving slower when observers are tracking it with their eyes than fixation (Aubert, 1886; von Fleischl, 1882). Our results suggest that eye movements including smooth pursuit and fixation affect visual motion prediction using a time‐to‐contact task, which is consistent with the Aubert–Fleischl phenomenon.

### 4.2 Influence of motion configuration on visual motion prediction and pursuit responses

An increase in pursuit latency, reductions in initial eye acceleration and steady‐state eye velocity were observed in the pursuit second‐order condition as well as previous studies (Churan & Ilg, 2001; Hawken & Gegenfurtner, 2001; Ilg & Churan, 2004; Lindner & Ilg, 2000; Miyamoto et al., 2020b). These reductions in smooth pursuit responses are in part due to poor retinal image motion induced by second‐order motion, which does not evoke explicitly optokinetic nystagmus (OKN) (Harris & Smith, 1992; Lelkens & Koenderink, 1984). In fact, since neurons in areas MT and MST are related to the direction of the retinal image motion, second‐order motion elicits a weaker response in areas MT and MST than that for first‐order motion (Albright, 1992; Churan & Ilg, 2001; Ilg & Churan, 2004; O’Keefe & Movshon, 1998).

Although the second‐order motion stimuli attenuated the pursuit response at all target velocities, the effect on visual motion prediction was not consistent. When the object velocity was 3 deg/s, the constant error for the second‐order motion shifted to an earlier response compared to the first‐order motion. In addition, the shift toward the early prediction time was correlated with a reduction in steady‐state eye velocity at 3 deg/s (*r* = 0.69), but such a relationship was not found at 4 and 5 deg/s. These results suggest that the effect of second‐order motion on visual motion prediction is dependent on the object velocity. This is comparable with previous studies reporting that perceived object velocity is consistent for object motion at or above 4 deg/s regardless of motion stimuli (Gegenfurtner & Hawken, 1996), which is termed as form‐cue invariance (Albright, 1992). Therefore, it is likely that velocity perception and visual motion prediction at relatively high velocity (≥ 4 deg/s) are not associated with smooth pursuit behavior.

This dissociation between smooth pursuit and motion perception seems curious because a number of studies have reported the relevance between smooth pursuit and motion perception such as the direction‐discrimination and perceived velocity of a moving object (Beutter & Stone, 2000; Krukowski & Stone, 2005; Stone & Krauzlis, 2003; Van Donkelaar et al., 2000; Watamaniuk & Heinen, 1999). However, it is also pointed out that although the MT and MST are involved in both smooth pursuit and motion perception (Newsome et al., 1989; Salzman et al., 1990), these cortical areas are not the final stages involved in motion perception (Ilg, 2008). In fact, this dissociation between eye movement and motion perception has been reported other than velocity perception (Spering, Pomplun, et al., 2011). Although the mechanism of the dissociation between smooth pursuit and visual motion prediction at object velocities of 4 and 5 deg/s is still uncertain, previous and our studies suggest that visual motion prediction at relatively high velocity is related to the velocity perception rather than the accuracy of smooth pursuit per se. As another possibility, catch‐up saccades were observed during the second‐order motion stimulus as well as previous studies (Churan & Ilg, 2001; Hawken & Gegenfurtner, 2001; Ilg & Churan, 2004; Lindner & Ilg, 2000). Recent studies have demonstrated that saccades affect the velocity perception of visual motion (Goettker et al., 2018; 2019). Therefore, saccadic eye movements during smooth pursuit could influence motion perception during tracking a moving object.

### 4.3 Aspect of accuracy of visual motion prediction

**FIGURE 8:**
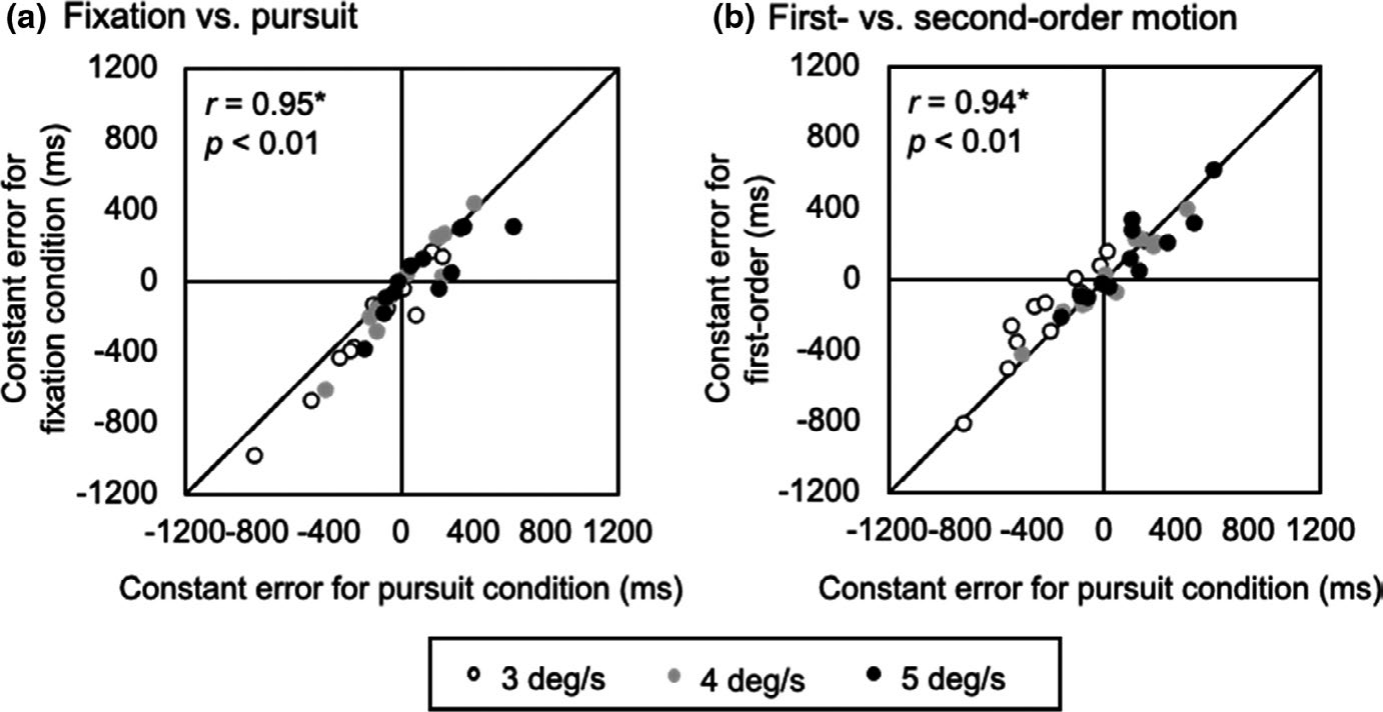
Relevance of the constant error of visual motion prediction between the conditions. (a) Comparison between the fixation and pursuit first‐order conditions. (b) Comparison between the pursuit first‐ and second‐order conditions. In each figure, open, gray, and black circles indicate 3 deg/s, 4 deg/s, and 5 deg/s conditions, respectively. The solid diagonal indicates the equality line

We attempted to determine which visual strategy is suitable for a better visual motion prediction based on the results of this study. Our results showed that smooth pursuit yielded the smaller absolute error at an object velocity of 3 deg/s, which indicates a better visual motion prediction in terms of accuracy during relatively lower target velocity. However, the difference in the absolute error between pursuit and fixation decreased with the increase in object velocity. Given that smooth pursuit always shifted the constant error to the late response regardless of the object velocity, the improved accuracy of visual motion prediction with pursuit may occur only at low object velocity in which the observers tend to make early estimations of visual motion prediction. The Pearson's correlation coefficients of the constant error showed quite high consistency within the observers regardless of the visual strategy and motion configuration (Figure 8), suggesting the subjective scale involved in visual motion prediction. Considering that the perception of time lapse is a subjective process even if the physical time is identical (Allman et al., 2014), it is likely that the observers estimate visual motion prediction by integration of their own internal clock and velocity perception. Therefore, we conclude that smooth pursuit affects visual motion prediction, while the accuracy of prediction depends not only on the visual strategy but also on individual‐specific perception of time.

## 5 CONCLUSION

Our findings expand previous studies regarding the influence of smooth pursuit on visual motion prediction. We attempted to determine whether smooth pursuit affects a temporal aspect of visual motion prediction using a time‐to‐contact task. Smooth pursuit led to a shift of the constant error to a later response compared to the fixation condition. Moreover, we attempted to apply the second‐order motion stimulus to the time‐to‐contact task during pursuit to clarify the influence of the motion configuration in visual motion prediction. The constant error for the second‐order motion shifted to an earlier response than the first‐order motion during pursuit when the object velocity was 3 deg/s. Therefore, our findings suggest that visual motion prediction is altered depending on the conditions of the eye movements and motion configuration for the moving object, such as second‐order motion.

## CONFLICTS OF INTEREST

The authors have no conflicts of interest to declare that are relevant to the content of this article.

## AUTHOR CONTRIBUTION

TM, KN, and SO conceived and designed research, TM and KN performed experiments, TM and KN analyzed data, TM, KN, YH, AK, KM, and SO interpreted results of experiments, TM and SO wrote manuscript, TM, KN, YH, AK, KM, and SO approved the final version of manuscript.

## Data Availability

The data that support the findings of this study are available from the corresponding author upon reasonable request.
